# Protective effect of *Aster tataricus* extract on NLRP3‐mediated pyroptosis of bladder urothelial cells

**DOI:** 10.1111/jcmm.15952

**Published:** 2020-10-08

**Authors:** Xin Wang, Ling Fan, Hao Yin, Yiqun Zhou, Xiaolong Tang, Xiaojun Fei, Hailin Tang, Juan Peng, Xiaoqin Ren, Yi Xue, Chunli Zhu, Jianping Luo, Qinglei Jin, Qingjiang Jin

**Affiliations:** ^1^ Department of Nephrology Suzhou Hospital of Integrated Traditional Chinese and Western Medicine Suzhou China; ^2^ Li Shicai School Inheritance Studio Suzhou Hospital of Integrated Traditional Chinese and Western Medicine Suzhou China; ^3^ Department of Pharmacy Suzhou Hospital of Integrated Traditional Chinese and Western Medicine Suzhou China

**Keywords:** ATE, interstitial cystitis, NLRP3 inflammasome, pyroptosis, urothelial cell

## Abstract

*Aster tataricus* L.f. is a traditional Eastern Asian herbal medicine used for the relief of uroschesis‐related illnesses and has been demonstrated clinically to exert satisfied effects. However, the mechanism of its therapeutic action remains unclear. The present study aimed to evaluate the protective mechanism of *Aster tataricus* extract (ATE) on CYP or LPS + ATP‐induced interstitial cystitis (IC), we successfully constructed the induced IC Sprague‐Dawley (SD) rat model and IC human urothelium cell (SV‐HUC‐1) model. The main compounds of ATE were determined by LC‐MS. After intervention, the changes on the bladder wall morphology and inflammation were observed in each group. SV‐HUC1 cell viability was measured by MTT and double stained with Hoechst 33342 and propidium iodide (PI). The expression levels of NLRP3, Pro‐caspase‐1, Caspsae‐1 p20, GSDMD, GSDMD‐N and Cleave‐IL‐1β in vivo and in vitro in different groups were detected by Western blotting. ATE significantly alleviated oedema and haemorrhage and reduced the inflammation index and histopathological score in SD rat bladder. The results of cell revealed that ATE could improve cell viability and decrease pyroptosis ratio. The expression of NLRP3 and other pyroptosis‐related protein was remarkably decreased by ATE both in vivo and in vitro. ATE may be used as an inhibitor of NLRP3 in treating IC. The discovery of NLRP3/Caspase‐1/GSDMD‐N as a new protective pathway provides a new direction for protecting cell against IC.

## 1 INTRODUCTION

Interstitial cystitis (IC) is a chronic condition characterized by chronic pelvic pain, pressure and/or discomfort perceived to be related to the urinary bladder.^1^ The pathogenesis of IC is not clear enough. At present, the main treatment methods for IC include oral medication, bladder perfusion, bladder hydraulic dilatation and urinary diversion. These treatments provide only partial relief after treatment, and there is no complete cure.^2^ Vera et al^3^ observed histopathology and found that the symptom of IC included vacuolization of urothelial cell, mucosal infiltration of lymphocyte, neutrophil and eosinophil granulocyte, as well as increase of mast cell numbers in all bladder wall compartments. The inflammatory response has played an important role in IC pathological physiology up to now.^4^

Nod‐like receptor protein 3 (NLRP3) inflammasome is closely related to the progression of IC, and NLRP3 signalling pathway plays a key role in the pathogenesis of bladder injury disease.^5^ NLRP3 inflammasome can be activated by a variety of substances, such as pathogen‐associated components, injury‐related molecules, environmental stimuli and pathogenic bacterium, and studies show that inflammatory markers such as NLRP3, Caspase‐1 and GSDMD in patients with IC are elevated.^6^, ^7^

*Aster tataricus* L.f. is the dry root and rhizome of Chinese herb A *tataricus*.^8^ *A tataricus* can be found in many regions in Eastern Asia including mainland China, South Korea and Japan.^9^ Research reports that *A tataricus* has been used for the relief of coughs and as an expectorant and it possesses diuretic, anti‐tumour and antibacterial activities.^10^ *A tataricus* extract (ATE) could suppress the activation of pro‐inflammatory cytokine and nuclear factor‐kappa B (NF‐κB) signalling pathway, so as to provide treatment for diabetic rat.^11^ A study outlining the use of a methanol extract of *A tataricus* exhibited significant inhibitory activity against the production of inflammatory cytokines (prostaglandin E2, interleukin‐6 and interleukin‐1 beta) and the expression of inflammatory enzymes (inducible nitric oxide synthase and cyclooxygenase‐2) via the inhibition of NF‐κB activation.^8^

In the present study, we explored the effect of ATE on experimental models of interstitial cystitis in vivo and in vitro. We hypothesized that ATE might suppress NLRP3 expression in the urothelial cells and reduce the IC symptom, and investigate the effect of ATE isolated from *A tataricus* on urothelial cells infected with interstitial cystitis (IC) and the therapeutic effects and possible mechanism of ATE on the IC, which could provide valuable evidence supporting its traditional use in the treatment of dysuria.

## 2 MATERIAL AND METHODS

### 2.1 Experimental objects

40 specific pathogen free (SPF) grade healthy female SD rats (180‐200 g in weight) were purchased from Qinglongshan Experimental Animal Center, Nanjing, China. Rats were housed in the feeding room with constant temperature (21‐23°C) and humidity (45%‐65%) and maintained in a 12 hours light‐dark cycle with free access to eat and drink. All experiments followed the National Institute of Health Guidelines for the Care and Use of Animals.

SV‐HUC‐1 cells used in this study were obtained from Nanjing University of Traditional Chinese Medicine. SV‐HUC‐1 were cultured in 37°C, 5% CO_2_ incubator (SANYO, XD‐101) with DMEM/F‐12K medium (Gibco, 31800‐105) containing 10% foetal calf serum (FBS, Lonsera, S711‐001S), 100 U/mL of Penicillin and 100 U/mL of Streptomycin (Beyotime, KGY002).

### 2.2 Preparation of ATE

After crushing, 0.5 kg *A tataricus* powder was poured into 95% ethanol for 1 hour and heat it at 90°C for 1 hour. After filtration, *A tataricus* fluid extract was evaporated into dried extract powder under vacuum of 60°C.^12^ The dried powder was dissolved in double‐distilled water for next experiments.

### 2.3 HPLC‐MS analysis of ATE

**Figure 1:**
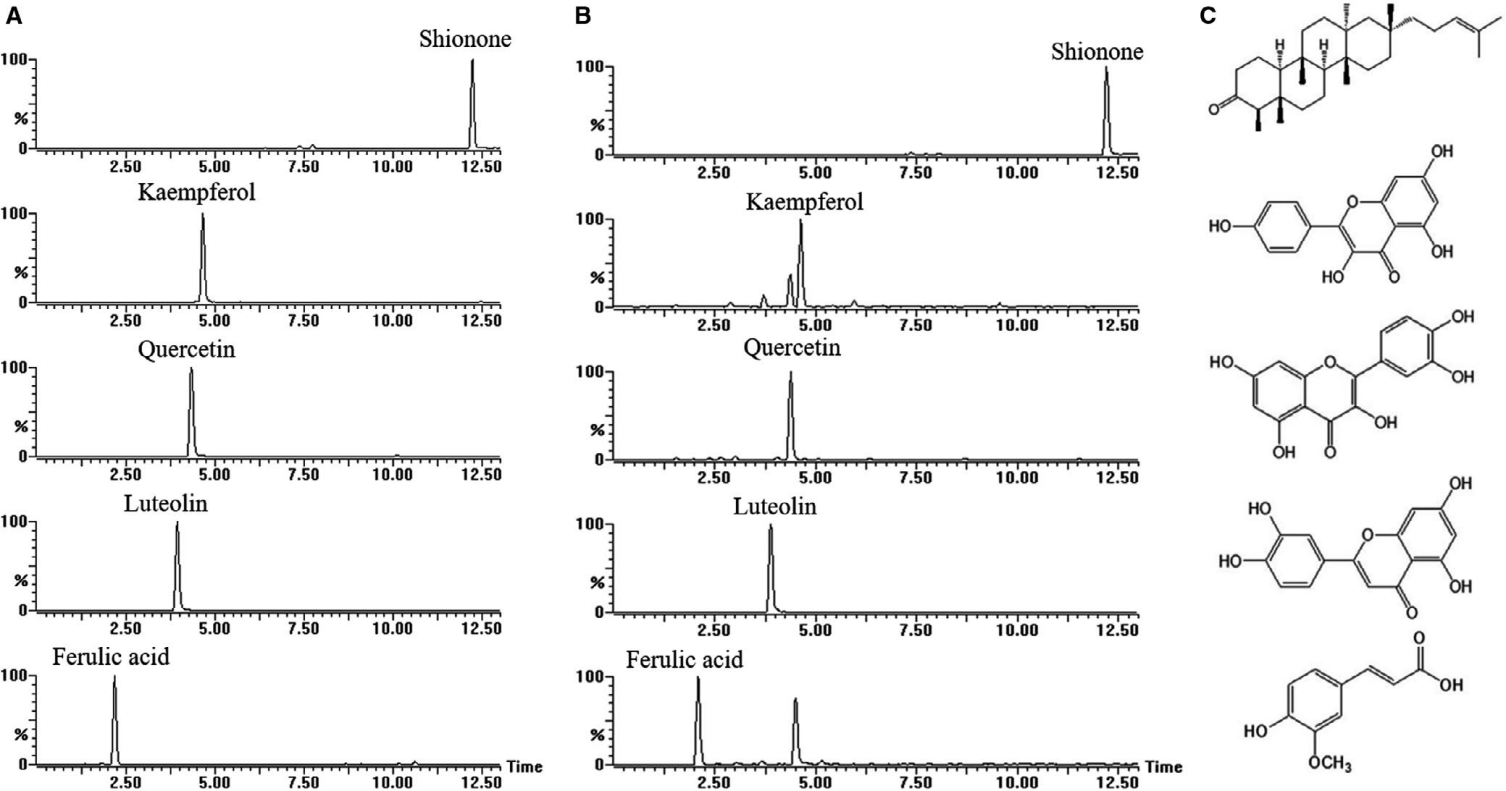
Representative extraction chromatograms of the five compounds. A, Standards. B, Samples. C, Chemical structures of five compounds in *A tataricus*

For quantitative determination of compounds, chromatographic analysis was carried out in a Waters Acquity HPLC system (Waters, Milford, MA, USA). The chromatographic separation was achieved at 30°C on an ACQUITY C18 column (100 × 2.1 mm, 1.7 μm). The mobile phases consisted of A (water) and B (acetonitrile) both containing 0.1% formic acid and used a gradient elution of 30%‐80% B at 0‐9 minutes, 80%‐95% B at 0‐13 minutes. MS was performed on a Xevo^™^ TQ‐S system from Waters (USA) tandem quadrupole mass spectrometer using an ESI source operated in positive (Shionone) or negative‐ion (others) mode. All the peaks of target compounds in the solution of *A tataricus* samples were unambiguously identified by the comparison of retention time, parent and product ions with standards. The product ions scan spectra are shown in Figure 1, and the concentrations of Shionone, Kaempferol, Quercetin, Luteolin, Ferulic acid were 3.78 mg/g, 0.34 mg/g, 3.98 × 10^−3^ mg/g, 1.02 × 10^−1^ mg/g, 3.12 × 10^−2^ mg/g, respectively. All instrumentations were controlled and synchronized by MassLynx data systems (version 4.1) from Waters.

### 2.4 IC rat model

After 1 week of adaptive feeding, animal modelling was carried out according to Table 1 below. Rats were divided into five groups with 8 in each group: control (no‐CYP treatment + saline), saline (CYP treatment + saline), ATE‐1.2 (CYP treatment + ATE‐1.2, 1.2 g/kg bodyweight), ATE‐2.4 (CYP treatment + ATE‐2.4, 2.4 g/kg bodyweight) and Me‐40 (CYP treatment + Me‐40, 40 mg/kg bodyweight).^13^ On the 9th day, after 10% chloral hydrate anesthetized by intraperitoneal injection, the bladder tissue was removed, weighed and fixed; blood was taken from the abdominal aorta, and the serum was centrifuged and stored in the refrigerator at −80°C. All procedures of animal experiments in this study were in accordance with the Regulations of Experimental Animal Administration issued by the Ministry of Science and Technology of the People's Republic of China.

**Table 1:** CYP modelling and ATE treatment

Group (n = 8)	Day‐(1‐6)	Day‐7	Day‐8	Day‐9
Control (oral)	Saline	Saline	Saline	anaesthesia
Saline (CYP model)	Saline	CYP	Saline	anaesthesia
CYP + ATE‐1.2 (oral)	ATE	CYP + ATE	ATE	anaesthesia
CYP + ATE‐2.4 (oral)	ATE	CYP + ATE	ATE	anaesthesia
CYP + Me‐40 (injection)	Mesna	CYP + Mesna	Mesna	anaesthesia

Note

CYP was given intraperitoneal injection at a dose of 150 mg/kg; ATE‐1.2, ATE 1.2 g/kg; ATE‐2.4, ATE 2.4 g/kg; Me‐40, Mesna 40 mg/kg. After 48 h of modelling, the SD rats were anaesthetized, and the follow‐up experiments were carried out. (SD rat gavage: 1 mL/100 g; Intraperitoneal injection in SD rats: 0.3 mL/100 g).

### 2.5 Measurement of bodyweight, bladder wet weight, oedema and haemorrhage

Bodyweight and bladder wet weight of every rat from each group was measured by precision balance (Shimadzu, Japan). According to Gray's criteria,^14^ oedema and haemorrhage indicators were scored macroscopically: Oedema is considered severe when fluid is seen inside and outside the bladder wall (3+); Moderate (2+) was limited to the internal mucosa; Mild (1+) indicated between normal and moderate. The haemorrhage score was as follows: There was a blood clot in the bladder (3+); Mucosal haematoma (2+); Telangiectasia or vesical vasectasia (1+); And normal (0).

### 2.6 Histopathological assay

The weight of bladder tissue was weighed firstly, and pathological analysis of the bladder tissues after infection was carried out. The paraffin sections prepared for haematoxylin‐eosin (H&E) staining by slicer (Histocore Biocut, Laica). The histological changes of the stained sections were observed using a light microscope equipped with a digital camera (Olympus, Japan) to observe the morphology of the cells, so as to evaluate bladder lesions, interstitial inflammation, oedema and mast cell infiltration. Histopathological score: normal epithelial cells, no inflammatory cell infiltration and ulcer (0); Epithelial cells decreased, submucosal oedema flattened, mild haemorrhage, a little ulcer (1+); Mucosal erosion, inflammatory cell infiltration, fibrin deposition, bleeding, multiple ulcers and other serious changes (2+).

### 2.7 Western blotting assay

The steps of protein extraction and Western blot analysis are referred to the previous literature.^15^ In brief, the extracted bladder protein tissue was loaded into a 10% SDS‐polyacrylamide gel and transferred into a PVDF membrane after electrophoresis. After incubating with primary and secondary antibodies and washing (NLRP3, ASC, Pro‐caspase‐1, Caspsae‐1 p20, GSDMD, GSDMD‐N and Cleave‐IL‐1β, Affinity; Goat Anti‐Rabbit IgG (H + L) HRP, Affinity), the ECL Plus assay Kit (Affinity, K002) was used for colour rendering. The gel analysis system scanned each strip protein, and the grey value of the strip was measured by image analysis software (Image J).

### 2.8 Immunofluorescence assay

The 4 μm sections of bladder tissues were dehydrated with 100%, 90%, 70% and 50% ethanol for 5 minutes, respectively. The samples were heated until boiled twice in a water bath to recover the antigen and then cooled down at room temperature for 3‐4 hours. Afterwards, tissue samples were dipped in 0.5% Triton X‐100 for 2 minutes at room temperature. The cells were fixed with 4% paraformaldehyde for 20 minutes, permeabilize with 0.5% Triton X‐100 for 20 minutes and blocked with 5% BSA blocking solution for 60 minutes at room temperature followed by washing with PBS. Thereafter, the samples were incubated with 1:200 dilution of primary antibodies (Rabbit anti‐Mouse antibody) for overnight at 4°C. Following overnight incubation, cells were washed three times with PBS and incubated for 2 hours in dark with second antibody (Goat Anti‐Rabbit, IgG (H + L) HRP). Later, cells were washed with PBS, the excess buffer was removed, and ECL chemiluminescence solution (Affinity, USA) was added. Representative fluorescence images were obtained using fluorescence microscope (Olympus, Japan), and positive staining was recorded.

### 2.9 *Modelling and administering of SV*‐*HUC*‐*1 cells*

After 2 hours administration with different concentrations of ATE, 10 μg/L LPS and 2.5 mmol/L ATP were added to stimulate the cells for 12 hours.^16^ The drug was administered in the following groups, as shown in Table 2.

**Table 2:** ATE treatment in different groups

Group	Medium	LPS	ATP	ATE
Control	+	—	—	—
IC model	+	+	+	—
25‐ATE	+	+	+	25 μg/mL ATE
50‐ATE	+	+	+	50 μg/mL ATE
100‐ATE	+	+	+	100 μg/mL ATE

### 2.10 Assays cell activity by MTT and Hoechst33342 and PI double staining

The SV‐HUC‐1 cells were placed in a 96‐well plate at a density of 1 × 10^4^ cells/well, respectively. Different concentrations of ATE were added into medium, and samples were incubated for 24 hours. After incubation, the cell cultures were mixed with MTT assay reagent for 4 hours and then read on microspectrophotometer (Nanodrop, Thermo, USA). After treatments, the cells in each group were stained with 5 mg/mL Hoechst 33342 and 10 mg/mL PI for 10 minutes in the dark. The stained cells were observed using fluorescence microscope (Olympus, Japan). The apoptotic cells were stained by Hoechst 33342 (blue) and pyroptosis cells by PI (red).

### 2.11 Infection with overexpression (NLRP3) lentivirus vectors

The recombinant lentivirus vectors for NLRP3 and empty vector were provided by Genechem (Shanghai, China). The 293T cells overexpressing NLRP3 were transfected by HiTransG P (Genechem, Shanghai, China), and the grouping was shown in Table 3. The infection efficiency could be observed by fluorescence microscope at 72 hours after infection.

**Table 3:** The grouping case of overexpression

Group	Lentivirus empty vector	Lentivirus overexpression (NLRP3)	LPS + ATP	ATE (100 μg/mL)
1	+	−	−	−
2	−	+	−	−
3	−	−	+	−
4	−	+	+	−
5	+	−	+	+
6	−	+	+	+

### 2.12 Data analysis

Statistical analyses were performed using one‐way ANOVA and were expressed as means ± standard error of the mean (SEM), followed by the Tukey‐Kramer test. *P* < 0.05 was considered statistically significant.

## 3 RESULTS

### 3.1 Component test of ATE

This study established and verified a UPLC‐MS method for simultaneous determination of ATE (Figure 1A,B), and chemical structures of the five compounds in *A tataricus* were shown in Figure 1C.

### 3.2 ATE ameliorated bladder damage induced by CYP

**Figure 2:**
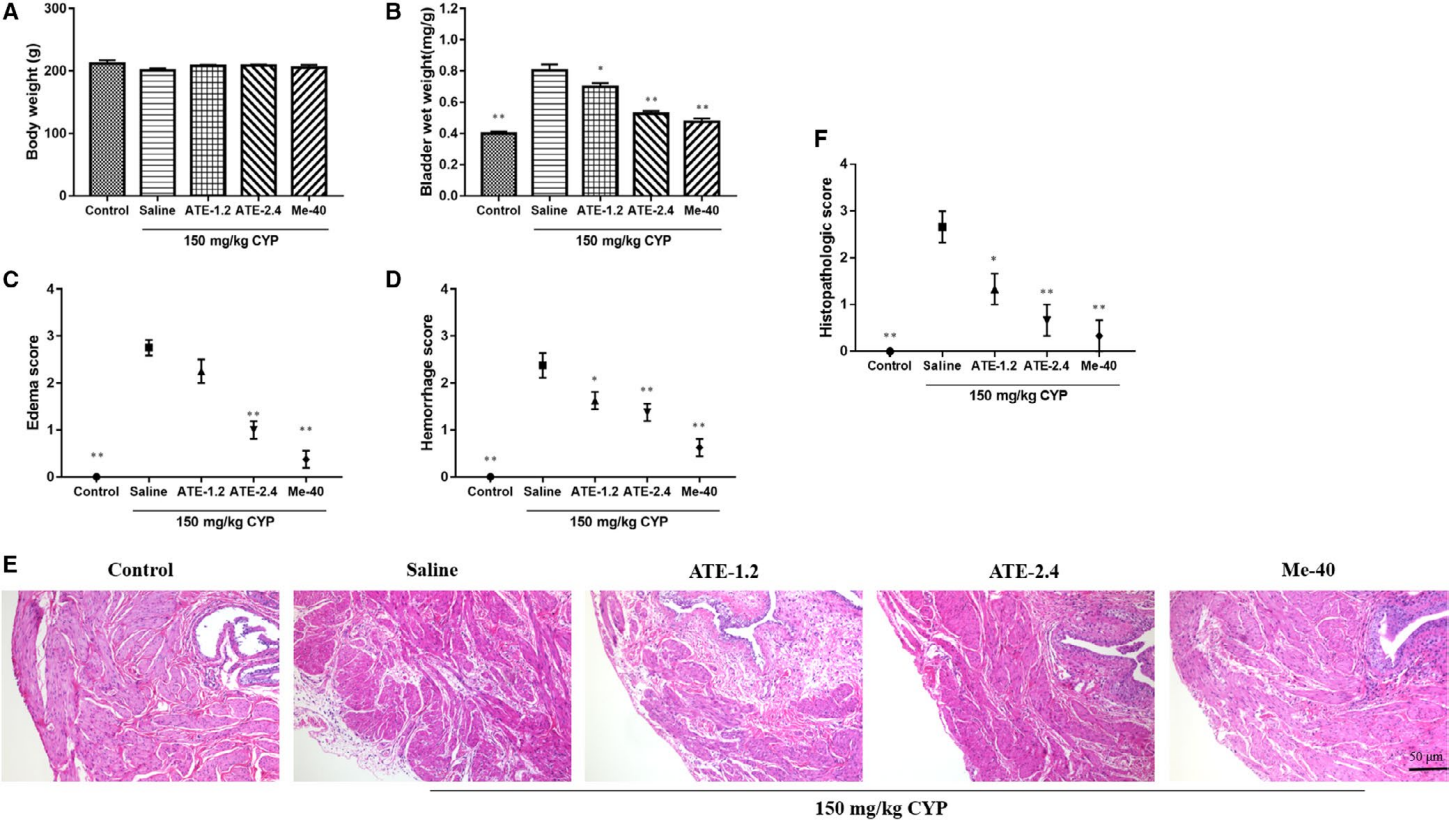
Effect of treatment with ATE on macroscopic evaluation of the bladder. A, Bodyweight. B, Wet weight of bladder. C, Oedema score of bladder was evaluated in CYP‐induced interstitial cystitis in mice. D, Haemorrhage score of bladder was evaluated in CYP‐induced interstitial cystitis in mice. E, Bladder sections were stained with haematoxylin and eosin (H&E). F, Histological evaluation was performed according to Gray's criteria. All values are expressed as mean ± SEM (n = 8 per group). *P < 0.05, **P < 0.01 compared with CYP group

There was no significant difference of bodyweight in control, saline and experimental groups (*P* > 0.05, Figure 2A). However, the wet weight of bladder in control group were significantly lower than that in saline (CYP‐induced) group; Compare with the saline group, the wet weight of bladder in each medicated group decreased significantly (*P* < 0.05) to some extent, and bladder wet weight of ATE‐2.4 was lower than that of ATE‐1.2 (Figure 2B). In Figure 2C,D, we found that in the macro score of bladder oedema and haemorrhage, the score of the saline group was generally about 2‐3 points, while the score of each medicated group recovered to 0‐2 points. The results suggested that CYP‐induced modelling had been successful and the ATE could relieve the symptom of oedema and haemorrhage.

The urinary epithelium, lamina propria and muscularis of the control group were normal. The histological changes of saline group included oedema, vasodilatation, haemorrhages status and showed infiltration of inflammatory cells. In the medicated groups, the bladder sections still showed oedema, vasodilatation, and inflammation, but less severe than the model group (Figure 2E). A conclusion was obtained that the medicated groups had lighter symptoms of inflammation and the ATE‐2.4 showed better improvement than the ATE‐1.2 in CYP‐induced (Figure 2E,F).

### 3.3 ATE reduce the expression of pyroptosis‐related protein in bladder

**Figure 3:**
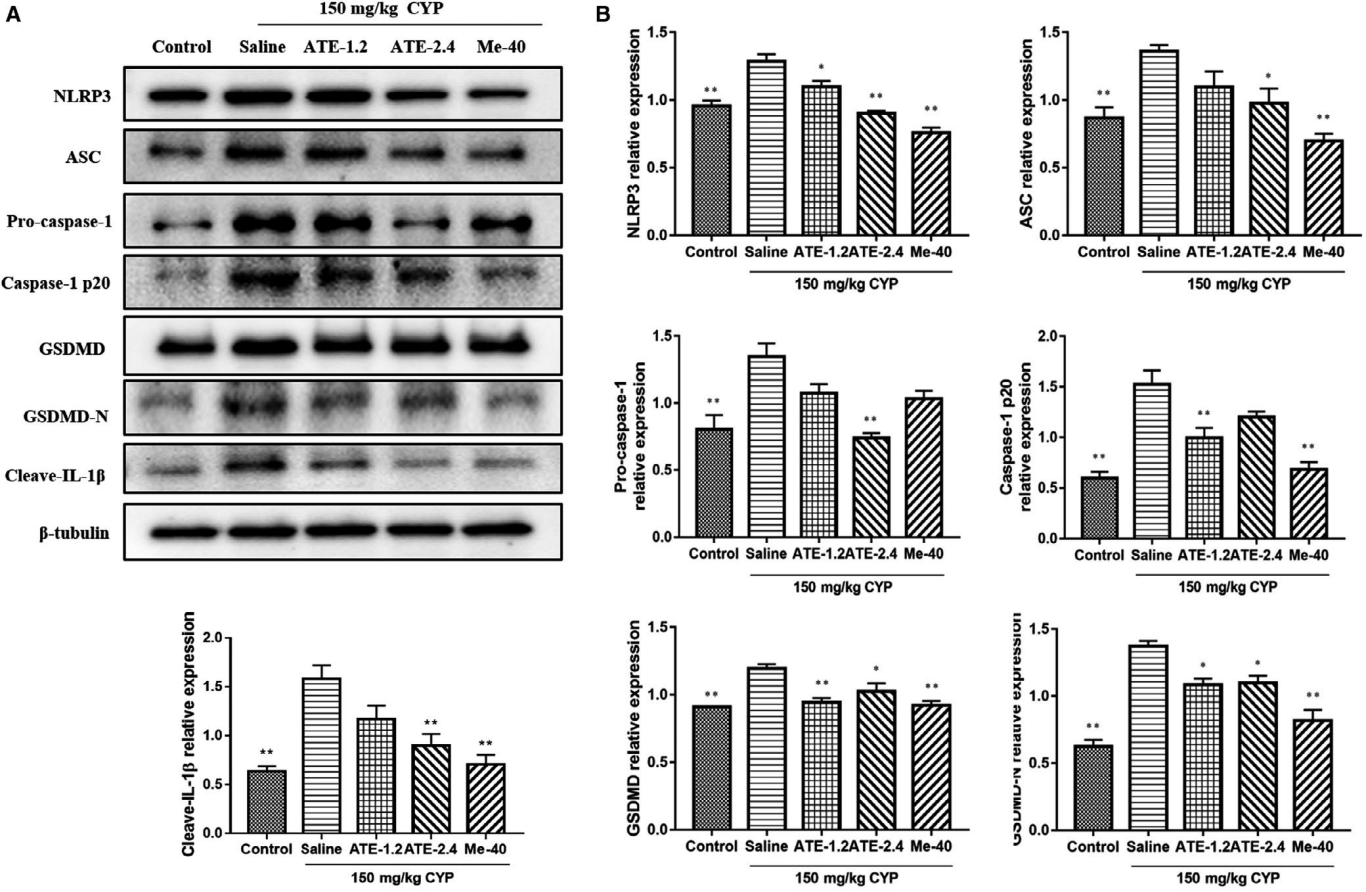
Effect of ATE on the pyroptosis‐related proteins of bladder in mice with CYP‐induced interstitial cystitis. A, Western blot analysis of the expression of pyroptosis‐related proteins in bladder. B, Densitometric analysis of pyroptosis‐related proteins relative expression. All values are expressed as mean ± SEM (n = 3 per group). *P < 0.05, **P < 0.01 compared with CYP group

The relative expression of NLRP3, ASC, Pro‐caspase‐1, Caspase‐1 p20, GSDMD, GSDMD‐N and Cleave‐IL‐1β was detected by Western blotting. Compared with control group, the relative expression of NLRP3, ASC, Pro‐caspase‐1, Caspase‐1 p20, GSDMD, GSDMD‐N and Cleave‐IL‐1β in saline (CYP model) group was higher; The expression of NLRP3, ASC, Pro‐caspase‐1, Caspase‐1 p20, GSDMD, GSDMD‐N and Cleave‐IL‐1β in medicated groups was lower than that in saline group; As the concentration of ATE increases, the relative expression level of NLRP3, ASC, Pro‐caspase‐1 and IL‐1β was decreased (*P* < 0.05, Figure 3A,B).

### 3.4 ATE ameliorated SV‐HUC‐1 cells damage induced by ATP + LPS

**Figure 4:**
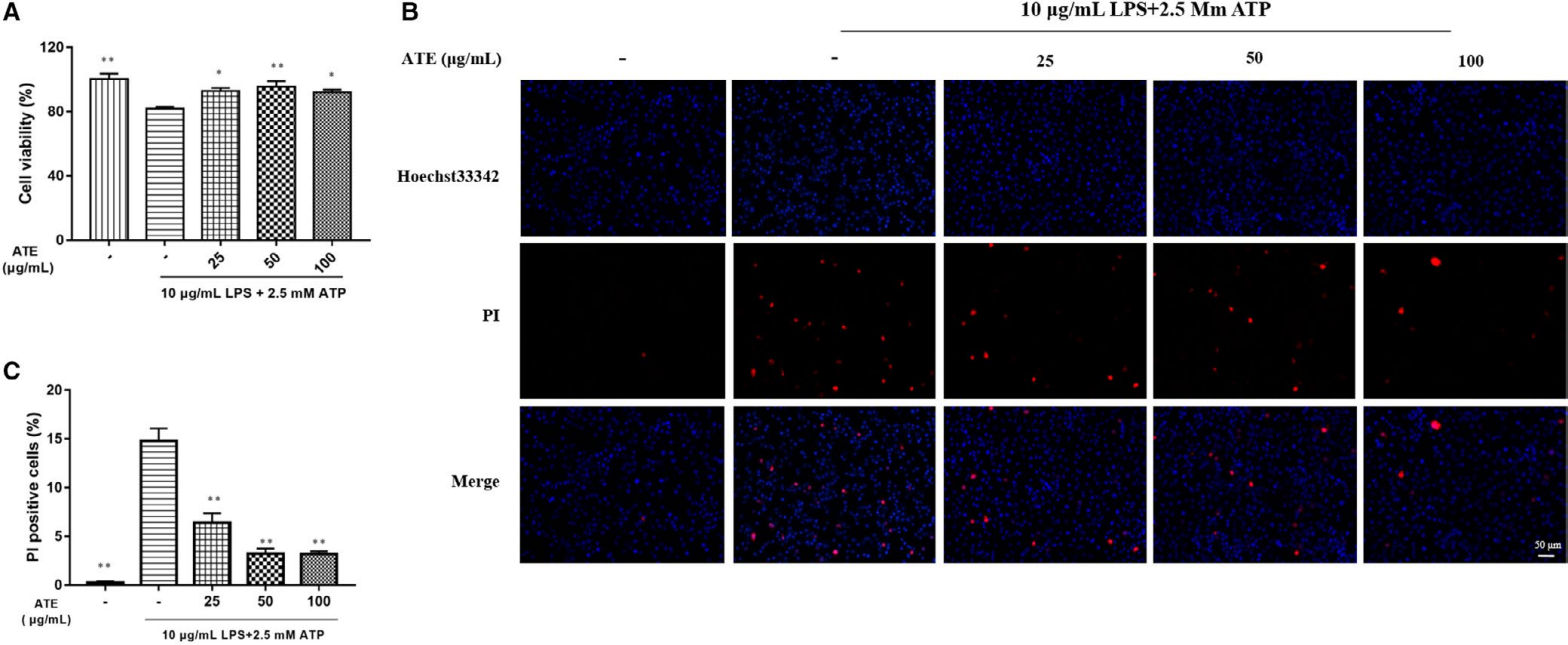
Effect of ATE on the LPS/ATP‐induced pyroptosis of SV‐HUC1 cells. A, Cell viability was measured by MTT. B, Representative images of PI‐positive cells. C, The calculated percentage of PI‐positive cells. All values are expressed as mean ± SEM (n = 3 per group). *P < 0.05, **P < 0.01 compared with LPS/ATP group

We measured the cell viability of SV‐HUC1 cells which were modelled with LPS/ATP and incubated with different concentrations of ATE. The cell viability of ATE medicated groups showed little differences compared with control group (in Figure 4A). We detected the degree of pyroptosis of urothelial cells using Hoechst33342 + PI double staining method. As shown in Figure 4B, there were more PI‐positive cells in IC model (no ATE) group compared with control and ATE medicated groups (*P* < 0.01, Figure 4C).

### 3.5 ATE reduce the expression of pyroptosis‐related protein in cell

**Figure 5:**
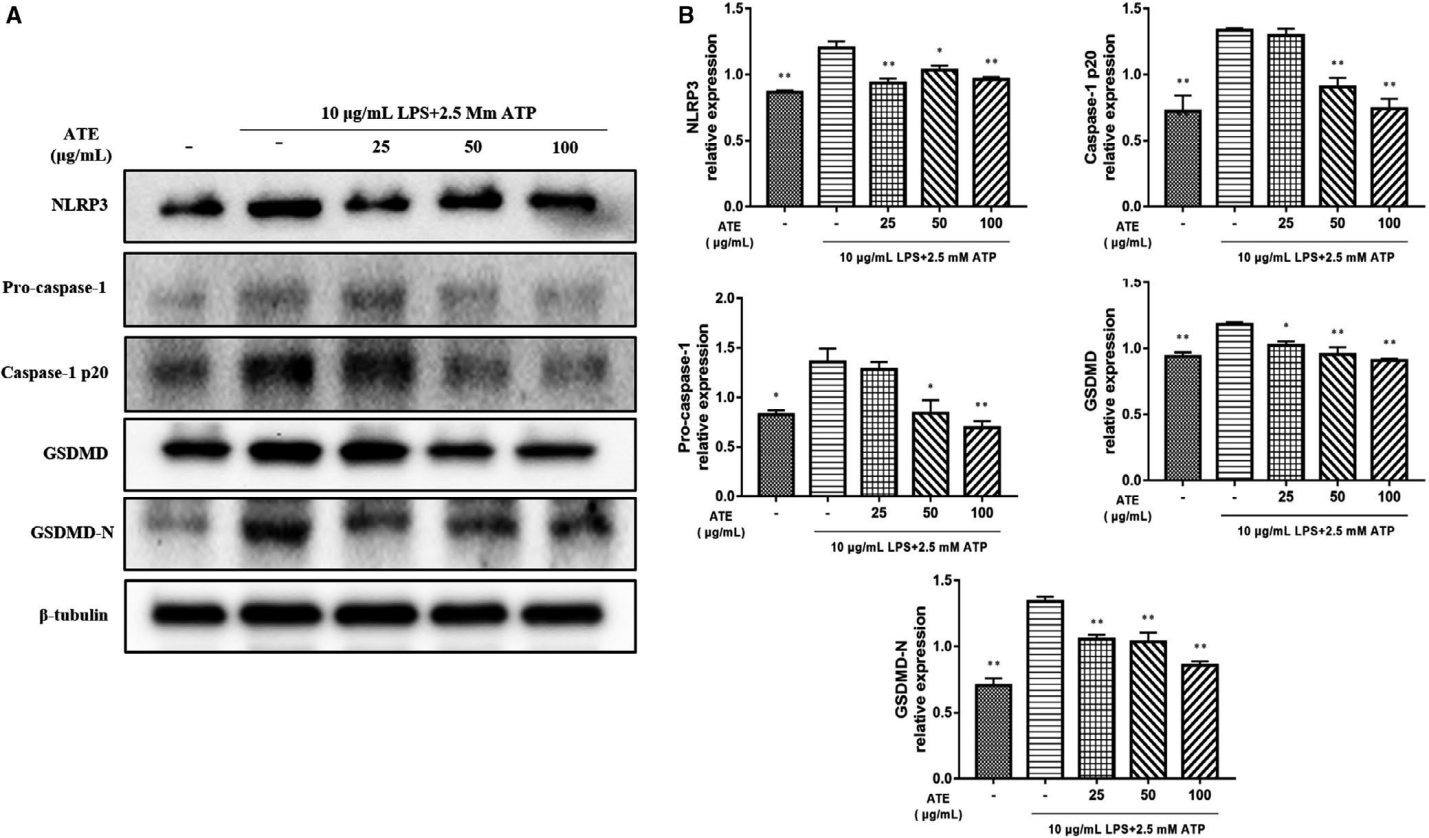
Effect of ATE on the proteins in SV‐HUC‐1 cells with LPS/ATP‐induced pyroptosis. A, Western blot analysis of the expression of pyroptosis‐related proteins. B, Densitometric analysis of pyroptosis‐related proteins relative expression. All values are expressed as mean ± SEM (n = 3 per group). *P < 0.05, **P < 0.01 compared with LPS/ATP

The expression of NLRP3, Pro‐caspase‐1, Caspsae‐1 p20, GSDMD, and GSDMD‐N in SV‐HUC‐1 cells in different groups was detected by Western blotting. In Figure 5B, the expression of NLRP3, GSDMD and GSDMD‐N in ATE medicated groups was decreased compared with that in no medicated group, and the GSDMD‐N group decreased most significantly (*P* < 0.01, Figure 5A,B).

### 3.6 *Immunofluorescence analysis of NLRP3 in bladder* in vivo*and vitro*

**Figure 6:**
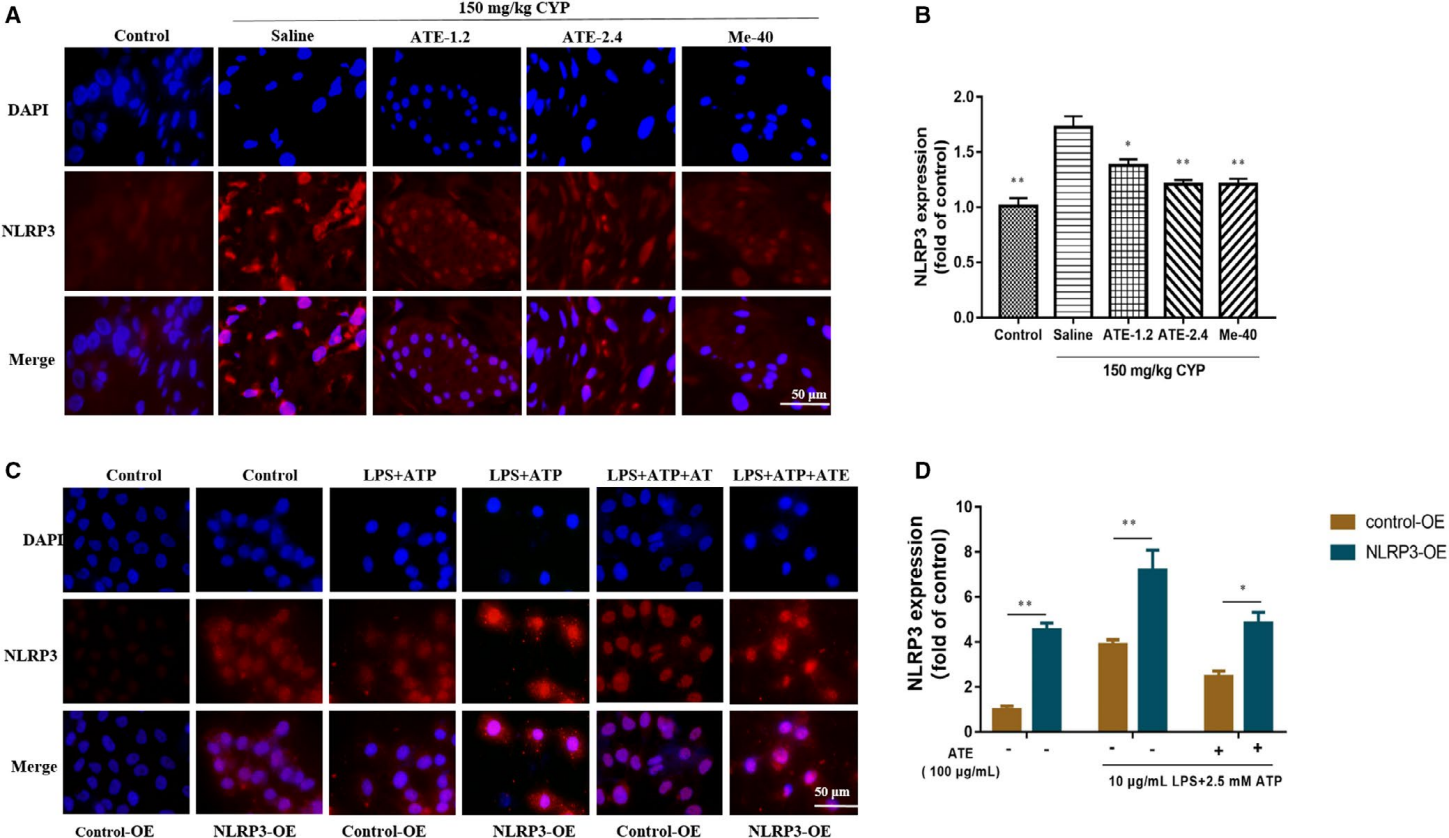
Immunofluorescence analysis of NLRP3 in vivo and in vitro. A, Fluorescence images of NLRP3 expression in bladder sections. B, Fluorescence intensity analysis of NLRP3 expression. C, Fluorescence images of NLRP3 expression in SV‐HUC‐1 cells. D, Fluorescence intensity analysis of NLRP3 expression. All values are expressed as mean ± SEM (n = 3 per group). *P < 0.05, **P < 0.01

The bladder tissues were observed under a fluorescence microscope, results showed that significant fluorescence signals were detected in the CYP‐induced groups compared with the control group, and the NLRP3 fluorescence signals of ATE‐1.2 and ATE‐2.4 group was significantly lower than that of the saline group, respectively (*P* < 0.05, *P* < 0.01, in Figure 6A,B).

In order to investigate the mechanism of NLRP3 in pyroptosis signalling pathway, we over‐expressed NLRP3 protein in SV‐HUC1 cells. Results showed that significant fluorescence signals were detected in the LPS + ATP‐treated groups compared with the control group, but the NLRP3 expression level of the medicated groups was significantly lower than that of the LPS + ATP‐treated groups; At the same time, the expression (NLRP3) of LPS + ATP‐treated groups had increased compared with that of no‐LPS + ATP‐treated groups, and the expression (NLRP3) of ATE medicated group was lower than that of no medicated group (Figure 6C,D).

### 3.7 *Effect of ATE on the pyroptosis‐related proteins in SV*‐*HUC1 cells*

**Figure 7:**
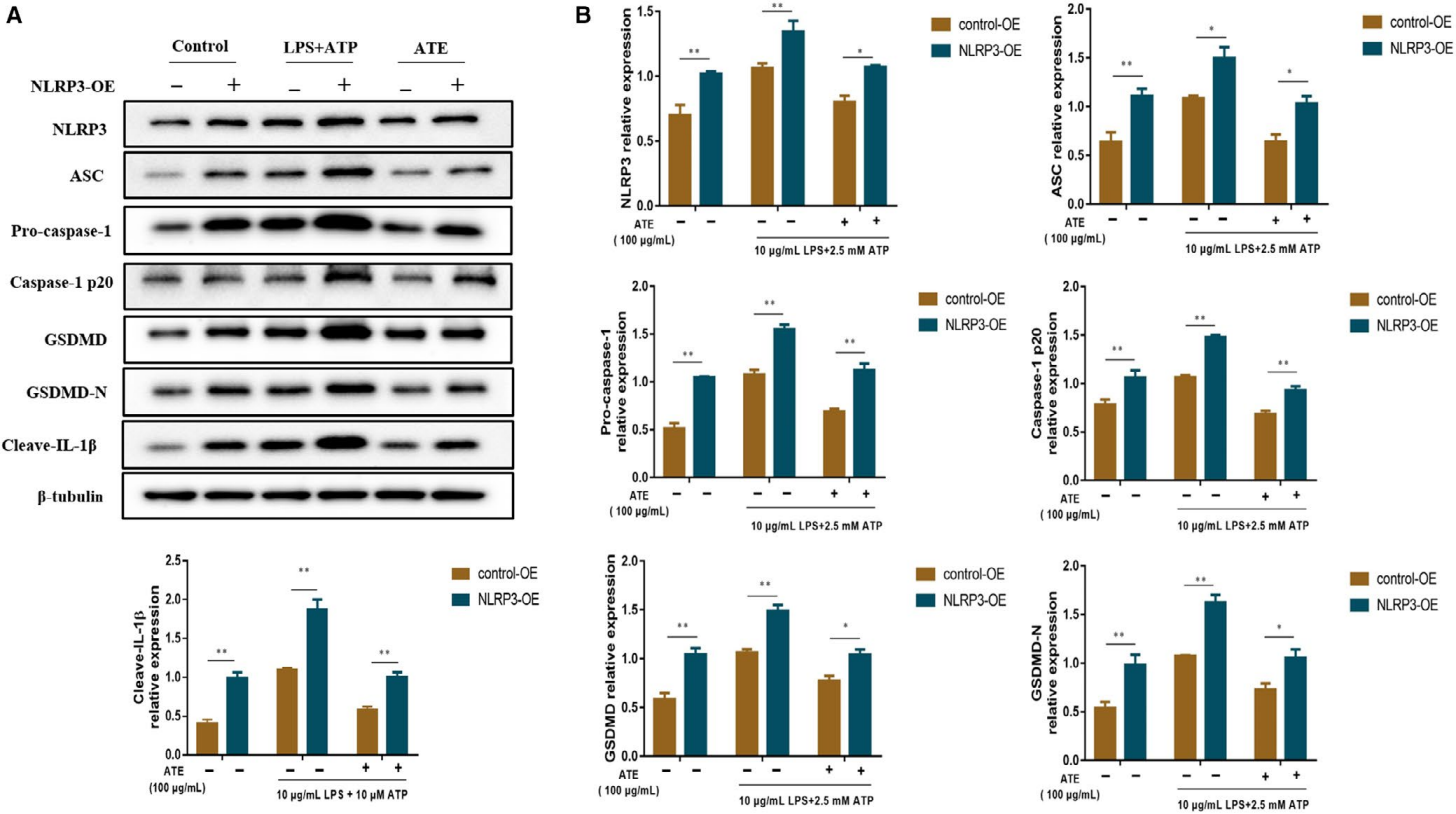
Effect of ATE on the proteins in SV‐HUC‐1 cells with LPS/ATP + NLRP3‐OE‐induced pyroptosis. A, Western blot analysis of the expression of pyroptosis‐related proteins. B, Densitometric analysis of pyroptosis‐related proteins relative expression. *P < 0.05, **P < 0.01

In Figure 7A, the relative expression levels of pyroptosis‐related proteins were detected by Western blotting. The expression levels of NLRP3, ASC, Pro‐caspase‐1, Caspsae‐1 p20, GSDMD, GSDMD‐N and IL‐1β in NLRP3‐OE groups were increased significantly compared with that in control‐OE groups (*P* < 0.05), and ATE‐medicated groups showed lower expression level compared with no medicated groups (Figure 7B).

## 4 DISCUSSION

The cause of IC is still unclear, because its treatment success rate is not high and easy to relapse, which is still challenging in clinical practice. The inflammation is one of the important factors of IC.^17^ The present study investigated the potential therapeutic effects of ATE on IC. We found ATE has a therapeutic effect on CYP‐induced rat IC and LPS + ATP‐induced cell IC. ATE treatment reduced the bladder inflammation in IC rats and alleviated the pyroptosis in SV‐HUC‐1. Furthermore, ATE exerted its therapeutic effect on IC by inhibiting NLRP3‐GSDMD pathway activation.

Pyroptosis‐inflammation plays an important role in the development of IC.^18^ Study has shown that inflammatory markers such as NLRP3, Pro‐caspase‐1, Caspase‐1, GSDMD and GSDMD‐N in patients with IC are elevated.^19^ Our study found that the expression of NLRP3, Pro‐caspase‐1, Caspase‐1, GSDMD and GSDMD‐N of IC model was decreased after ATE treatment compared with the untreated group. The main histological changes of IC caused by CYP in rat were bladder mucosal bleeding and submucosal oedema,^20^ mucosal bleeding and oedema were improved, and bladder inflammation was alleviated in IC rat after ATE treatment (Figure 3). The main pathological feature of IC was the loss of bladder urothelial cell integrity,^21^ and the viability of SV‐HUC‐1 cell was improved, and ratio of pyroptosis was decreased compared with that of IC model after ATE treatment (Figure 4).

Pyroptosis, which is also known as cellular inflammatory necrosis, induces the release of cellular contents to activate the inflammatory response.^22^ Previous studies demonstrated that the high expression of Caspase‐1 and GSDMD was an indicator of pyroptosis.^23^, ^24^ The inhibition caused by small molecule is more efficient than single blockade of other components (ASC, Caspase‐1) or its downstream IL‐1β, suggesting that NLRP3 is a central molecule in the NLRP3‐Caspase‐1‐IL1β pathway.^25^ NLRP3 recruits and activates Caspase‐1 after the identification of pathogen molecular pattern, cleaves Gasdermin D (GSDMD) to form GSDMD‐N and induces cell membrane perforation, cell rupture, release of contents, causing pyroptosis.^26^, ^27^ Correspondingly, we confirmed that the activation of NLRP3 inflammasome could induce pyroptosis, which was demonstrated by the lysis of GSDMD, an increase in GSDMD‐N content, and the presence of PI‐positive cells. ATE administration could inhibit the expression of NLRP3 and relieve the symptoms of IC in vivo and in vitro.

GSDMD is a pyroptosis‐inducing factor, which leads to physical rupture of the cell membrane that mediates release of matured IL‐1β from the cell and plays a role in driving pyroptosis induced by the NLRP3. Caspase‐1 cleaves GSDMD between the Asp276 and Gly277 and generates an N‐terminal and a C‐terminal fragment, the expression of N‐terminal fragment of GSDMD alone induced pyroptosis, whereas the C‐terminal fragment provided autoinhibition prior to cleavage of the full‐length protein.^28^ GSDMD‐N is an inflammasome‐associated and pyroptosis‐related signalling molecule. Consistent with the results of previous study,^28^ we found that rat and cell IC had a significant increase in the expression of GSDMD and GSDMD‐N, and ATE treatment had atherapeutic effect on reducing NLRP3 and GSDMD‐N (Figure 4 and Figure 7). Taken together, our results indicate that ATE confers a protective effect on bladder injury and improves cell viability via suppressing the expression of pyroptosis‐related proteins and indicating NLRP3/GSDMD‐N pathway.

Mesna, was positive medicine used for IC treatment, the continuous delivery of Mesna provides a constant source of thiol groups available to the bladder to bind acrolein and reduce local injury.^29^ Previous study had reported the anti‐inflammatory activity of *A tataricus* extract, and *A tataricus* could be used as a diuretic in clinic and has a good therapeutic effect.^30^ In present study, five important active components have been determined in aster, including shionone, luteolin, quercetin, kaempferol and ferulic acid. Shionone in *A tataricus* significant expectorant and antitussive effects, which is the characteristic component of *A tataricus*. Luteolin and quercetin are also expectorants and antitussive drugs.^10^ For example, kaempferol and quercetin have significant effects on inhibition of lipid peroxidation reactivity and immunoregulation, ferulic acid can scavenge free radicals, antibacterial and anti‐inflammatory.^31^ Compared with Saline, ATE was significantly improved in bladder macroscopic evaluation and cell viability. High doses have a better protective effect than low doses in vivo and in vitro, but the protective effect still not as good as Mesna.

Consistent with previous studies, our study indicates that NLRP3 plays a critical role in pyroptosis via NLRP3/GSDMD pathway.^32^, ^33^ ATE administration blocked the CYP‐induced bladder inflammation and inhibited NLRP3 and GSDMD‐N expression in the bladder and urothelial cell. We speculated that ATE could significantly inhibit the expression levels of NLRP3, Caspase‐1 and GSDMD in the NLRP3 inflammatory pathway. Therefore, our results show that the anti‐inflammatory effect of ATE on IC is achieved by inhibiting NLRP3/GSDMD‐N inflammatory pathway.

In conclusion, our results suggest that ATE has a potential protective effect against CYP or LPS + ATP‐induced IC in vivo or in vitro. ATE exhibited anti‐inflammatory properties in the bladder of rat and urothelial cell via suppressing the expression of pyroptosis‐related protein and down‐regulating the NLRP3/GSDMD‐N signalling pathway. These findings suggest a potential clinical benefit of NLRP3 targeted pharmacotherapy and ATE treatment for bladder inflammatory conditions.

## CONFLICT OF INTEREST

The authors report no conflicts of interest in this work.

## AUTHOR CONTRIBUTIONS

**Xin Wang:** Data curation (equal); Writing‐original draft (lead). **Ling Fan:** Formal analysis (equal). **Hao Yin:** Formal analysis (equal). **Yiqun Zhou:** Investigation (supporting). **Xiaolong Tang:** Methodology (supporting). **Xiaojun Fei:** Methodology (equal). **Hailin Tang:** Project administration (lead). **Juan Peng:** Project administration (supporting). **Xiaoqin Ren:** Resources (supporting). **Yi Xue:** Software (supporting). **Chunli Zhu:** Validation (equal). **Jianping Luo:** Writing‐review & editing (equal). **Qinglei Jin:** Writing‐review & editing (supporting). **Qingjiang Jin:** Conceptualization (lead).

## Data Availability

Data will be available from the corresponding author upon reasonable request.
